# Supervised learning in spiking neurons

**DOI:** 10.1186/1471-2202-14-S1-P159

**Published:** 2013-07-08

**Authors:** Charlotte Le Mouel, Pierre Yger, KD Harris

**Affiliations:** 1 Institute of Neurology, Departement of Neuroscience, Physiology and Pharmacology, University College London; 2 Ecole Normale Superieure, Paris

## 

A critical function of the brain is to learn behavioral responses appropriate to given sensory stimuli. In some cases, the appropriate response to a stimulus is explicitly provided. For example in classical conditioning, after pairing of an unconditioned stimulus that naturally drives a certain behavioral response with a second conditioned stimulus that is originally neutral, the animal learns to later respond to produce the behavior in response to the conditioned stimulus.

In statistics and machine learning, the problem of associating a particular class of inputs with an externally specified target output is called "supervised learning". Various statistical techniques have been developed for supervised learning, for example the support vector machine (SVM) which has seen success in a wide variety of applications. However, the way supervised learning could occur in spiking neurons remains unclear. One potential answer to this question is provided by an algorithm termed the "Tempotron"[1], which has been successfully applied to real-world classification, but contains features unlikely to occur in real neurons: neurons are required to fire exactly one spike when a particular category is detected; furthermore the algorithm can only work reliably if input patterns are perfectly normalized.

We developed a robust algorithm for supervised learning of spatio-temporal patterns by spiking neurons receiving inputs through both excitatory and inhibitory synapses. By extending concepts from the SVM framework to spiking neurons, we generalized the Tempotron rule to mathematically derive a learning rule for classifying input patterns. In this framework, each target category is represented by a pool of output neurons that are required to together fire several spikes in response to a correct pattern, but not to fire otherwise. Performance is assessed by a hinge-loss error function on the summed activity of the pool. We suggest a candidate cellular mechanism for the rule, in which a training signal arriving when behavior is performed operates to consolidate an eligibility trace left by coincidence of presynaptic firing and postsynaptic depolarization.
